# Cancer of unknown primary derived from regressed breast cancer

**DOI:** 10.1007/s00432-024-05768-5

**Published:** 2024-05-04

**Authors:** Maria Pouyiourou, Theresa Mokry, Maximilian Feszler, Andrea Teifke, Andreas Kreft, Alwin Krämer

**Affiliations:** 1 Clinical Cooperation Unit Molecular Hematology/Oncology, German Cancer Research Center (DKFZ) and Department of Internal Medicine V, University of Heidelberg, Im Neuenheimer Feld 280, 69120 Heidelberg, Germany; 2 Department of Diagnostic and Interventional Radiology, University of Heidelberg, Heidelberg, Germany; 3 Department of Hematology and Stem Cell Transplantation, University Hospital Essen, University of Duisburg-Essen, Essen, Germany; 4 Department of Diagnostic and Interventional Radiology, University of Mainz, Mainz, Germany; 5 Institute of Pathology, University of Mainz, Mainz, Germany

To the editor

Cancer of unknown primary (CUP) is a heterogenous group of cancers for which the anatomical site of origin remains occult even after detailed investigations (Krämer et al. 2023). The unique biology of these tumors remains unknown. Distinct subsets of patients with CUP have been defined, with a minority of patients (15–20%) belonging to clinico-pathological subgroups with a more favorable prognosis, including women with isolated axillary lymph node metastases.

We here describe the case of a 46 year old, premenopausal woman, who was diagnosed with left-sided axillary lymph node metastases of a CK7 + , GATA3 + , Mammaglobin + , CK20-, ER-, PR-, HER2- adenocarcinoma with a proliferation rate of 30%, suggestive of triple-negative breast cancer, in August 2020 (Fig. 1). However, mammography, breast ultrasound and magnetic resonance imaging (MRI) of the breast did not show a breast tumor. Also, abdominal ultrasound, computed tomography of thorax and abdomen, MRI of pelvis, thorax and brain, and bone scintigraphy did not show signs of malignancy, leading to the diagnosis of favorable subtype breast-like CUP syndrome. Neither tumor cells nor blood-derived DNA revealed somatic or germline mutations predisposing to breast and ovarian cancer by whole exome sequencing.

**Fig. 1:**
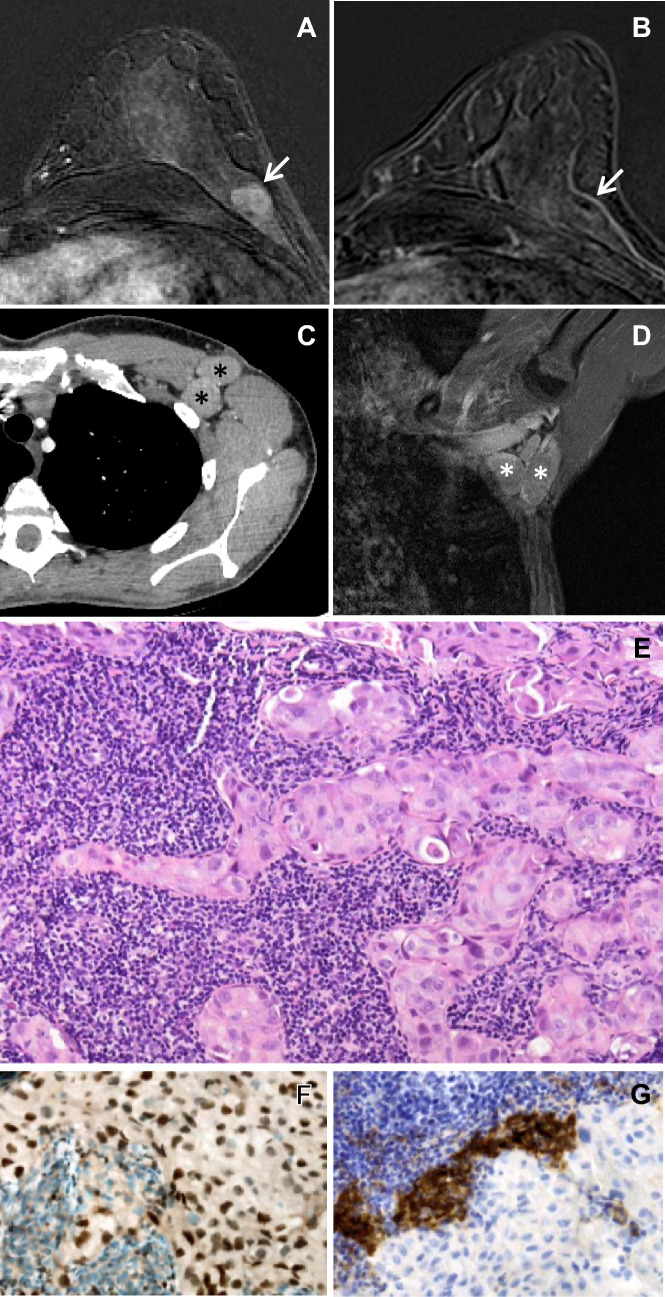
Imaging and histology of left breast and lymph node metastases in the left axilla. Gadolinium-enhanced magnetic resonance imaging (MRI) of the breasts from July 2019 and August 2020 is shown in Panels A and B, respectively. Whereas a lobulated 1 cm tumor in the upper outer quadrant of the left breast is clearly visible in the image from July 2019 (Panel A, arrow), no tumor is found in the corresponding images from August 2020 (Panel B). Panels C and D depict computed tomography (CT) and MRI from August 2020 of lymph node metastases in the left axilla (asterisks), respectively. Panels E, F, and G show hematoxylin–eosin (H&E) staining (400x), and GATA3 and programmed death ligand 1 (PD-L1) (each 400x) immunostainings of a CT-guided core needle biopsy from a lymph node metastasis of the left axilla from August 2020, respectively

Because of microcalcification in the right breast the patient had received yearly screening including mammography, breast ultrasound and MRI since 2011. Retrospectively, in a breast MRI from July 2019 a lobulated, 1 cm tumor in the upper outer quadrant of the left breast with early intensive gadolinium enhancement and subsequent rapid washout (Fig. S1), strongly suggestive of breast cancer, that was no longer detectable in the breast MRI from August 2020, had been overlooked, a finding further corroborating the breast origin of the perceived CUP syndrome.

Due to the assumed origin of breast-like CUP from an occult primary breast cancer, patients with breast-like CUP syndrome are managed according to the treatment protocols for nodal positive breast cancer. Accordingly, the patient received neoadjuvant chemotherapy with epirubicin/cyclophosphamide followed by paclitaxel/carboplatin, and had left-sided axilla dissection plus bilateral mastectomy on her own discretion in 2021. The mastectomy sample revealed no signs of malignant lesions. However, one remaining positive axillary lymph node was detected. The patient then received radiotherapy of the left-sided chest wall and axillary region followed by post-neoadjuvant chemotherapy with capecitabine and has been in complete remission since completion of the treatment.

Although it remains unclear why no primary tumor can be detected in CUP syndrome, immunological rejection has been discussed as a potential mechanism. Tumor-infiltrating lymphocytes are frequently found and associated with smaller tumor size and better prognosis in triple-negative breast cancer, a molecularly heterogeneous disease with poor outcome (Loi et al. 2019; Schmid et al. 2020). Interestingly, triple-negative breast cancer primary tumors contain more tumor-infiltrating lymphocytes and express lower programmed death ligand 1 (PD-L1) levels than lymph node metastases derived thereof, which indicates that immune escape plays a role in tumor progression (Ogiya et al. 2016; Li et al. 2018). Accordingly, lymph node metastases of our patient contained only few, strongly PD-L1-expressing tumor-infiltrating lymphocytes with a combined positive score (CPS) of 20%.

Together, this case represents a rare occasion to document that favorable subtype CUP syndrome of women with isolated axillary lymph node metastases may indeed derive from adenocarcinoma of the breast.

## Supplementary Information

Below is the link to the electronic supplementary material.

Supplementary file1 (DOCX 1340 KB)
