# Supplementary material for: Cancer of unknown primary derived from regressed breast cancer
Source: J Cancer Res Clin Oncol. 2024 May 4;150(5):229. doi: 10.1007/s00432-024-05768-5 (PMC11069480; doi:10.1007/s00432-024-05768-5)
Supplement: Supplementary file 1 — Supplementary file1 (DOCX 1340 KB) [file 432_2024_5768_MOESM1_ESM.docx]

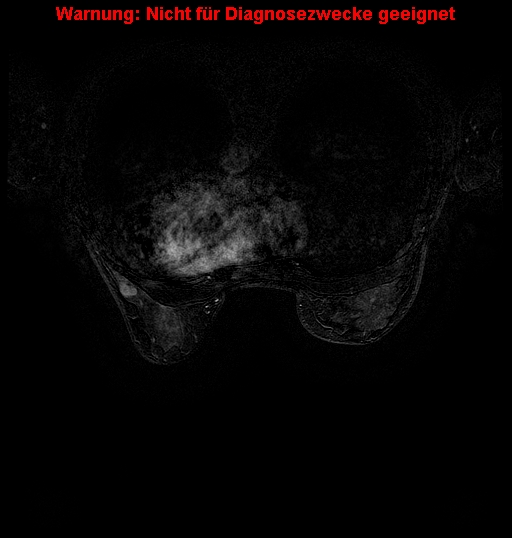

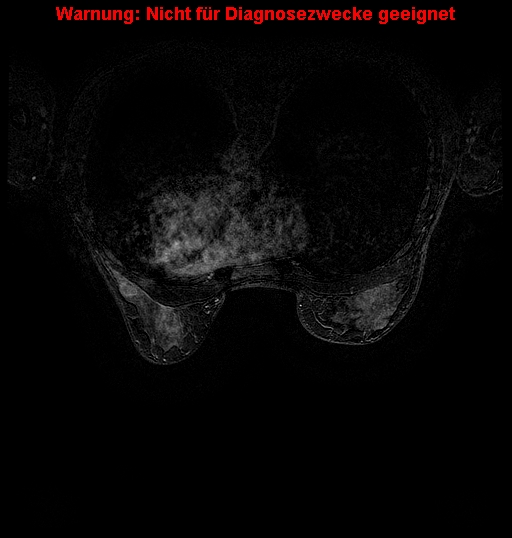

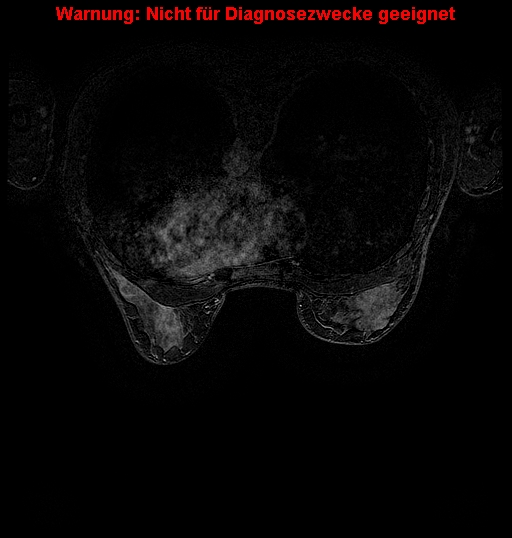

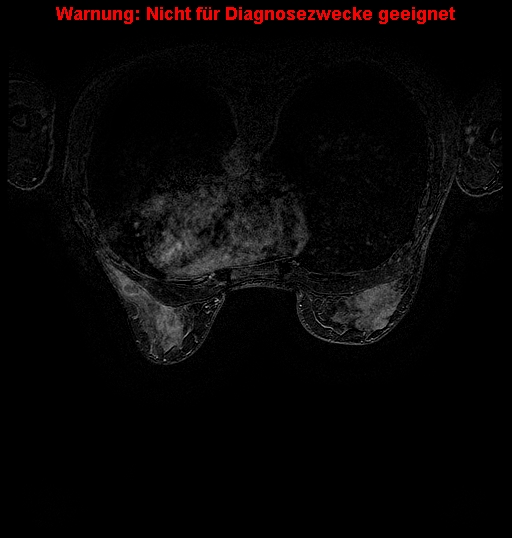


**A**

**B**

**C**

**D**

**Supplementary Figure S1.** Gadolinium-enhanced magnetic resonance imaging of the breasts from July 2019, showing gadolinium washout of the breast tumor (arrows) over time from Panel A to D.
